# How instructional leadership and creativity related factors shape students’ reading performance: evidence from a Bayesian multilevel mediation approach using PISA 2022 Türkiye data

**DOI:** 10.3389/fpsyg.2026.1907891

**Published:** 2026-09-17

**Authors:** Nermin Er Aydemir, Tuncer Fidan

**Affiliations:** 1 Department of Turkish and Social Sciences Education, Faculty of Education, Alanya Alaaddin Keykubat University, Alanya-Antalya, Türkiye; 2 Department of Educational Sciences, Faculty of Education, Alanya Alaaddin Keykubat University, Alanya-Antalya, Türkiye

**Keywords:** creativity, curiosity, imagination, instructional leadership, multilevel path analysis, PISA 2022 Türkiye, reading performance

## Abstract

**Introduction:**

This study aims to examine the influences of student-level creativity characteristics, school-level creativity-supportive conditions, and instructional leadership on reading performance.

**Methods:**

This study employed PISA 2022 data for Türkiye. A total of 7,250 students and 196 school principals’ data were analyzed by employing multilevel path analysis and Bayesian estimation. Missing data were addressed through Full Information Maximum Likelihood. We reported model fit indices in path analysis to validate our model. We conducted convergence checks, posterior predictive checks, and sensitivity analyses, and reported pooled values for the number of free parameters, the observed and replicated chi-square statistics, and the posterior predictive *p*-value (PPP) to validate our model in Bayesian estimation.

**Results:**

Our results revealed that at the student level, the effect of creativity and openness to intellect on reading performance is mediated by curiosity and imagination. At the school level, none of the indirect effects of instructional leadership on reading performance—through a creative school environment, a culture of openness, or creative activities—were found to be significant. Another notable mechanism is that socioeconomic and cultural status (ESCS) has a direct and positive association with reading performance. While creativity, intellectual openness, and imagination are associated with socioeconomic status, curiosity seems to function as a relatively egalitarian internal source of motivation not shaped by socioeconomic advantage. The pattern at the school-level presents a notably different picture. Our school-level findings revealed that instructional leadership had a negative direct relationship with reading performance. Furthermore, the strong direct association of academic school selectivity with reading performance revealed that exam-focused school stratification in Turkey is the dominant structural factor determining achievement.

**Discussion:**

Overall, this study revealed that the reading performance of Turkish students is shaped not directly from individual traits such as creativity–openness to intellect, but through motivational-cognitive mechanisms such as curiosity and imagination. The insignificance of the indirect paths of instructional leadership on reading through creative processes indicates that interventions aimed at improving reading should be designed not only around teaching cognitive skills but also around learning environments that spark curiosity and nurture imagination.

## 1 Introduction

Reading proficiency is one of the most important life skills. It is also one of the fundamental competencies that determines individuals’ ability to engage in lifelong learning, participate in social life, and contribute to economic productivity (OECD, 2019). Reading literacy is defined by the Programme for International Student Assessment (PISA) as “understanding, using, evaluating, reflecting on and engaging with texts to achieve one’s goals, to develop one’s knowledge and potential, and to participate in society” (OECD, 2019). Based on this definition, PISA acknowledges reading literacy as a broader set of competencies that enables students to engage with written information presented in texts for a specific purpose (OECD, 2019). Given its relevance for educational outcomes, researchers and policymakers should continue to focus on understanding the factors that support or impede reading achievement.

Türkiye has participated in PISA since 2003, and the country’s reading performance trajectory has experienced considerable ups and downs. Reading scores, which had reached their highest point in 2012, fell to their lowest in 2015 and then recovered partially to 466 in 2018 (OECD, 2014, 2016, 2019). In the most recent PISA exam in 2022, Turkish students scored 456 points in reading, which is 20 points below the OECD average of 476 (OECD, 2023a, p. 313). Türkiye is one of the countries whose PISA results have improved over a period of more than ten years in mathematics and science, while reading performance decreased by 10 points between 2018 and 2022 (OECD, 2023a). The patterns suggest that while there have been systemic improvements in other domains, reading literacy in Türkiye needs to be investigated further.

The COVID-19 pandemic led to a remarkable disruption of education systems across the world. Many countries switched to distance education during this period. The abrupt shift to distance education worsened the existing inequalities in terms of digital infrastructure, internet access, technological skills, parental support, and school type (OECD, 2023b). Research shows that the pandemic widened the educational gap between children based on their socio-economic status, creating a learning gap for disadvantaged students (Betthäuser et al., 2023). The average reading score in 2022 was a decline from the previous PISA exam for the OECD, which was the largest decline (OECD, 2023a). Therefore, it is important to identify the factors that can have a positive or negative impact on students’ reading performance to improve their reading skills.

The present study contributes to this line of inquiry in five respects. First, it draws on the newly introduced creativity module in PISA 2022, which provides an internationally comparable assessment of students’ creative dispositions and the extent to which their school environments foster creativity. In doing so, it addresses a specific theoretical and empirical gap in the relevant literature. Reading is increasingly theorized as a generative, meaning-constructing act rather than passive decoding (Zwaan, 2016). At the same time, both creativity-relevant dispositions and the leadership and climate conditions that cultivate them have been linked to achievement, but in isolated research streams. These streams have rarely converged on reading literacy specifically, and even less often within a single model that specifies how school-level leadership and climate conditions (Zhu et al., 2026) and student-level creative dispositions (Zhang et al., 2026) jointly shape reading outcomes. Establishing why these particular predictors should matter for reading, rather than for achievement in general, therefore motivates the theoretical framework developed below. Second, it focuses on Türkiye, an education system characterized by marked regional and school-type disparities and a recent downward trend in reading performance. Third, rather than examining student-level characteristics and school-level conditions separately, it integrates both within a single multilevel mediation model, thereby responding to calls in the leadership literature for greater ecological validity in school-effect research (Zhu et al., 2026). Finally, it employs a Bayesian estimation approach, which is particularly appropriate for multilevel mediation analyses with a relatively modest number of level-2 units (here, 196 schools) and for deriving credible intervals for indirect effects, where conventional frequentist methods such as the Sobel test are known to lack power and to assume symmetry (Miočević et al., 2018).

## 2 Theoretical underpinnings

Contemporary models of text comprehension frame reading as a constructive process in which the reader generates mental representations by integrating textual cues with prior knowledge and imagined referents. This constructive work is precisely the terrain of creativity-relevant dispositions: curiosity drives information search into the text, while imagination and adventurousness supply the simulative capacity that turns propositions into experienced scenes. Reading, in this sense, is a creative act at the cognitive level (Mar and Oatley, 2008). Within the situation model framework, comprehension corresponds to the unique “situations” described in the text: moving beyond surface form and the propositional text base, the reader builds a multidimensional mental world that integrates events, actors, and their spatial, temporal, causal, and intentional relations (Zwaan, 2016). Because constructing a situation model is a generative process rather than passive decoding, it provides the theoretical basis for linking creativity, curiosity, and imagination—together with the contextual conditions that nurture them—to reading comprehension (Zwaan, 2016).

Amabile's (1988) Componential Model of Creativity extends this rationale by positing that a creative act emerges from the interplay of individual and environmental factors. The creativity-relevant processes Amabile (1988) places at the model’s core map directly onto the student-level dispositions under investigation—creativity and openness to intellect, curiosity, and imagination and adventurousness—which govern the extent to which a person approaches a task flexibly, breaks out of established cognitive sets, and generates novel yet appropriate responses. Brought to bear on reading, these processes underpin the construction of a coherent situation model: curiosity supplies the motivational impetus to close informational gaps, imagination and adventurousness enable the mental simulation of events left unstated. Creativity and intellectual openness foster the integrative thinking required to connect textual elements (Zwaan, 2016). They also reinforce the search for novel ideas that sustain curiosity and expectations about possible outcomes that sustain imagination (Amabile and Pratt, 2016).

At the school level, these individual factors are enabled or inhibited by the social environment, which grounds the study’s contextual variables. Amabile and Pratt (2016) hold that environmental conditions (leadership, school environment and climate, and the curricular and extracurricular activities offered in schools) can either amplify or suppress a creative act, a proposition that aligns closely with the present study’s school-level constructs. Instructional leadership shapes the priorities, expectations, and pedagogical support that legitimize creative engagement. A creative school environment and climate establish the norms under which students find it safe and valuable to think divergently and to approach texts inquiringly. Creativity-oriented curricular and extracurricular activities, nurtured by a creative school environment and climate, provide the concrete opportunities through which creativity-relevant processes are exercised. Together, these conditions constitute the environmental support that allows individual creative dispositions to flourish rather than remain latent (Amabile and Pratt, 2016).

In his Instructional Leadership Theory, Hallinger (2011) proposes that school principals can positively influence classroom practices in two primary ways: by providing direct instructional support to teachers, or by fostering a professional learning community that emphasizes collaborative and innovative teaching practices to enhance student achievement. Through the latter mechanism in particular, principals acting as instructional leaders can cultivate a collaborative climate in which both teachers and students are motivated to implement innovative ideas. In such a climate, principals are expected to provide institutional support for school activities and extracurricular programs that nurture creativity. This clarifies why instructional leadership is investigated as a predictor of reading specifically rather than of achievement broadly. Instructional leadership is not assumed to act on comprehension directly, but to shape the school-level environmental conditions that determine whether students’ creative dispositions are exercised on tasks such as reading (Bellibaş et al., 2026). Reading performance thus serves as the outcome through which the downstream, creativity-mediated influence of leadership can be detected, which is precisely why the two sets of predictors are modeled jointly rather than in isolation (Zhang et al., 2026; Zhu et al., 2026).

## 3 Conceptual framework

Reading involves the processes by which individuals understand, interpret, and analyze written texts. This process develops students’ language skills, reading comprehension abilities, and thinking capacities (Zwaan, 2016). Reading comprehension, one of the aspects of reading, is defined by the National Institute of Child Health and Human Development (2000) as a fundamental skill for both students’ academic success and lifelong learning and is widely regarded as a comprehensive and multidimensional process of constructing meaning from texts (Mar and Oatley, 2008). It is considered a fundamental cognitive skill that contributes to children’s academic achievement and supports their participation in various aspects of adult life (Dollinger, 2016). Reading is not merely the process of decoding written symbols; rather, it is a process through which individuals construct meaning, interpret, think critically, make inferences, and develop their cognitive world through texts. This process was shaped by student- and school-level factors, which were elucidated in the following sections (Zwaan, 2016).

### 3.1 Student-level factors

As proposed in the Situation Model of Comprehension (Mar and Oatley, 2008; Zwaan, 2016) and the Componential Model of Creativity (Amabile, 1988; Amabile and Pratt, 2016), student-level factors like creativity and openness to intellect, curiosity, imagination and adventurousness are expected to enhance students’ reading performance. Predisposition to creativity and openness to intellect (CREATOP) is conceptualized as the fundamental characteristic of creativity-relevant processes, whereas curiosity (CURIOAGR) and imagination and adventurousness (IMAGINE) represent the functionalized subdimensions in which creative processes are transformed into behavior (Amabile and Pratt, 2016; OECD, 2024a).

In line with the assumptions of Amabile and Pratt (2016), Zwaan (2016), and Dollinger (2016) found that individuals’ creativity and intellect were positively related to their reading habits and comprehension levels. Creativity and openness to intellect manifest as divergent and associative thinking—the capacity to generate multiple interpretations and to link distant ideas—which is precisely the mental operation a reader performs when integrating events, actors, and their causal and intentional relations (Zwaan, 2016). Empirically, the intellectual aspect of openness is a reliable predictor of reading achievement, operating in part through cognitive ability and the disposition to seek out and reason about complex information (Zhang et al., 2026).

In addition, curiosity has been defined as a drive for information in the absence of extrinsic reward and has been acknowledged as a crucial motivation fostering educational attainment (Markey and Loewenstein, 2014). In a study conducted by Gurning and Siregar (2017), students’ achievement in reading comprehension with high curiosity was found to be higher than that of those with low curiosity, confirming the Componential Model of Creativity. The Gurning and Siregar (2017) also point out that students with high levels of curiosity demonstrate significantly higher reading comprehension skills than those with low levels of curiosity. Curious students are more active in the learning process, are more eager to tackle assigned reading tasks, and are not discouraged by having to try again and again to achieve the best possible understanding of what they read (Gurning and Siregar, 2017). Students with a strong sense of curiosity show greater interest in novel ideas, have a greater capacity for understanding, are more inclined toward intellectual exploration, possess a more inquisitive mindset, and are expected to have higher reading performance (OECD, 2024b). Curiosity was, therefore, identified as a powerful resource in increasing reading achievement in schools and decreasing dropout rates (Singh and Manjaly, 2022). Crucially for the present mediation logic, curiosity is not merely a correlate of reading achievement but the motivational mechanism that translates a general disposition toward openness into the active information-seeking a text demands. This is because creativity and openness to intellect describe a broad readiness to engage cognitively rather than a specific reading behavior. Their influence on reading performance is expected to be carried by curiosity, which supplies the concrete drive to close the informational gaps a situation model requires (Markey and Loewenstein, 2014; Zwaan, 2016). Empirical support for treating curiosity as a mediator rather than only a predictor comes from evidence that epistemic curiosity accounts for part of the relationship between openness to experience and learning outcomes (Hassan et al., 2015), which directly justifies its position between creativity–openness and reading performance in the present model.

Comprehension theorists have long argued that narratives function as simulations of the social world, such that readers actively imagine story events, characters, and settings rather than merely decoding propositions (Mar and Oatley, 2008). This simulative work is what allows a reader to fill the spatial, temporal, and causal gaps that a text does not make explicit, generating the inferences on which a coherent situation model depends (Zwaan, 2016). Indeed, the capacity to draw inferences is itself grounded in the imagination of alternative possibilities, so that imaginative and inferential processes operate in concert during reading comprehension (Mar and Oatley, 2008). Empirical evidence supports this link: across multiple experiments with hundreds of readers, mental-imagery ability and adventurousness were found to be reliably associated with stronger reading performance and richer engagement (Suggate and Lenhard, 2022). This body of work grounds the selection of imagination and adventurousness as a mediator. The effect of a general creativity-openness disposition on reading comprehension should operate through the specific simulative act of imagining unstated events, because it is imaginative elaboration, not creativity or openness in the abstract, that produces the inferences a coherent situation model depends on (Mar and Oatley, 2008; Zwaan, 2016). Imagination is thus theorized as a proximal cognitive process that channels the more distal trait of creativity–openness into reading performance, rather than as an independent predictor acting in parallel (Suggate and Lenhard, 2022).

According to the Situation Model Framework (Mar and Oatley, 2008; Zwaan, 2016) and the Componential Model of Creativity (Amabile and Pratt, 2016), the influence of creativity and openness to intellect on comprehension, however, is not only direct but is also channeled through the more proximal dispositions of curiosity and imagination. Theoretically, openness/intellect describes a broad tendency toward cognitive engagement, encompassing lower-order facets such as intellectual curiosity, imagination, and adventurousness (Amabile and Pratt, 2016). Because these facets are constituent expressions of the same underlying trait, more open and creative students are those most inclined to become curious about, and to imaginatively elaborate upon, what they read (Zwaan, 2016). Empirical work substantiates this generative chain in both directions. On the one hand, epistemic curiosity has been shown to mediate the relation between openness to experience and reading performance, indicating that open individuals learn more effectively in part because their openness fuels a motivation to seek out and resolve informational gaps (Hassan et al., 2015). Consistent with this, Ay and Soyer (2025) documented positive associations between openness and imagination, while Cavicchi (2024) demonstrated that curiosity enhances imagination and the willingness to explore novel ideas, collectively pointing to parallel and serial mediation pathways through curiosity and imagination. At this point, Suna et al. (2020) identified socioeconomic status as one of the main factors shaping student achievement and student-related variables in the Turkish context. The Suna et al. (2020) emphasized that there is a learning and talent gap between high- and low-group students.

Taken together, this evidence provides the empirical basis for the specific mediating structure tested here. Creativity and openness to intellect are theorized to influence reading not directly but by activating curiosity and imagination. These two are the proximal, functionalized expressions of the same underlying disposition (Amabile and Pratt, 2016). Curiosity and imagination have, in turn, been independently linked to reading comprehension (Gurning and Siregar, 2017; Suggate and Lenhard, 2022). Selecting curiosity and imagination as mediators is therefore not arbitrary but follows from converging theoretical and empirical evidence that they are the mechanisms through which a broad creative-open disposition is converted into the active, inference-rich processing that reading comprehension requires.

### 3.2 School-level factors

School-level conditions can shape individual creativity and academic outcomes. School leadership has a significant effect on features of the school organization, which positively influences the quality of teaching and learning (Leithwood et al., 2020). School principals’ efforts to improve teaching and learning have also been linked to school effectiveness and student achievement (Bellibaş et al., 2026). Schools that provide creative learning environments, stimulate students’ curiosity, and incorporate more creative activities into their instruction can have a positive impact not only on students’ creative development but also on a range of student outcomes, including reading skills. However, in the literature, the mechanisms through which school-level conditions supporting creativity influence reading achievement—particularly within multi-level frameworks that simultaneously examine processes at both the student and school levels—have not been sufficiently investigated. In particular, no study has been found that examines the relationship between creativity and reading using a multi-level model (student + school) in the Turkish context. Although the link between creativity and education is gaining increasing recognition (e.g., Li et al., 2023), a significant gap persists in the literature.

The school-level variables, correspondingly, pertain to the social environment component of Amabile's (1988) Componential Model of Creativity. Instructional leadership (INSTLEAD) is regarded as the principal organizational mechanism that shapes the creativity-supportive social climate of the school, functioning through the principal’s roles in defining the school mission, managing the instructional program, and fostering a positive learning climate (Hallinger, 2011). We hypothesized instructional leadership’s effect on reading to operate through four specific pathways at the school level: the creative school environment (CREENVSC), openness culture and climate (OPENCUL), creative school activities offered (ACTCRESC), and creative extracurricular activities (CREACTIV). Consistent with the Componential Model of Creativity (Amabile, 1988) and the Instructional Leadership Theory (Hallinger, 2011), instructional leadership is expected to influence reading achievement primarily indirectly, through these four school-level environmental mechanisms (Zhu et al., 2026).

The relevant literature presents empirical evidence for the hypothesized school-level relationships examined in this study. To illustrate, instructional leadership is positively related to the establishment of a school environment that supports creativity (Paletta and Alimehmeti, 2026) and to an open culture and climate (Qadach, 2026), both of which were identified as factors enhancing creative school/classroom practices and extracurricular activities (Paletta and Alimehmeti, 2026). Furthermore, school administrators’ and teachers’ willingness to contribute to a collaborative school environment that encourages risk-taking and the implementation of innovative ideas to enhance students’ reading achievement was found to be positively related to school climate openness (Barnová et al., 2022; Zhang et al., 2026). Considering this empirical evidence, we argue that teachers who are willing to implement innovative ideas to nurture student creativity (CREENVSC), and students who are willing to embrace cultural diversity and innovative practices (OPENCUL), constitute a basic precondition for the implementation of creative school activities (ACTCRESC) and creative extracurricular activities (CREACTIV) (Zhu et al., 2026). At this point, Guest (2018) emphasizes that the influence of extracurricular activities in exploring and developing student talent becomes stronger in schools with greater resources, more opportunities, and a wider range of formal activities, such as art, music, drama, and sports. This empirical finding implies that creative extracurricular activities can transmit the influence of creative school activities to students’ reading achievement. Finally, DiMartino and Jessen (2016) argue that selective schools could provide relatively more efficient school-level services, such as leadership, culture, and curricular and extracurricular activities, as teachers in these schools work with selected high-performing students with relatively higher reading performances.

These arguments are consistent with a growing body of evidence suggesting that school leadership influences student achievement primarily through indirect pathways. Instructional leadership, for instance, has been shown to exert its benefits on student outcomes via school climate and teacher-related mediators, with direct effects often weak or non-significant (Hallinger, 2011). Extending this logic to the present framework, the effect of instructional leadership on reading achievement is hypothesized to unfold as a sequential mediation. Leadership first shapes a creative and open school environment (CREENVSC, OPENCUL), which enables creative school and extracurricular activities (ACTCRESC, CREACTIV) (Bellibaş et al., 2026), and these activities ultimately transmit their influence to students’ reading achievement (Zhu et al., 2026).

By integrating individual creative processes and the surrounding school-level environmental mechanisms within a single theoretical framework, the Situation Model of Comprehension (Mar and Oatley, 2008; Zwaan, 2016), the Componential Model of Creativity, and the Instructional Leadership Theory (Hallinger, 2011) support the multilevel mediated structure of the present study (Figure 1). Students’ creative qualifications and the school environment that fosters them together shape reading performance.

**Figure 1:**
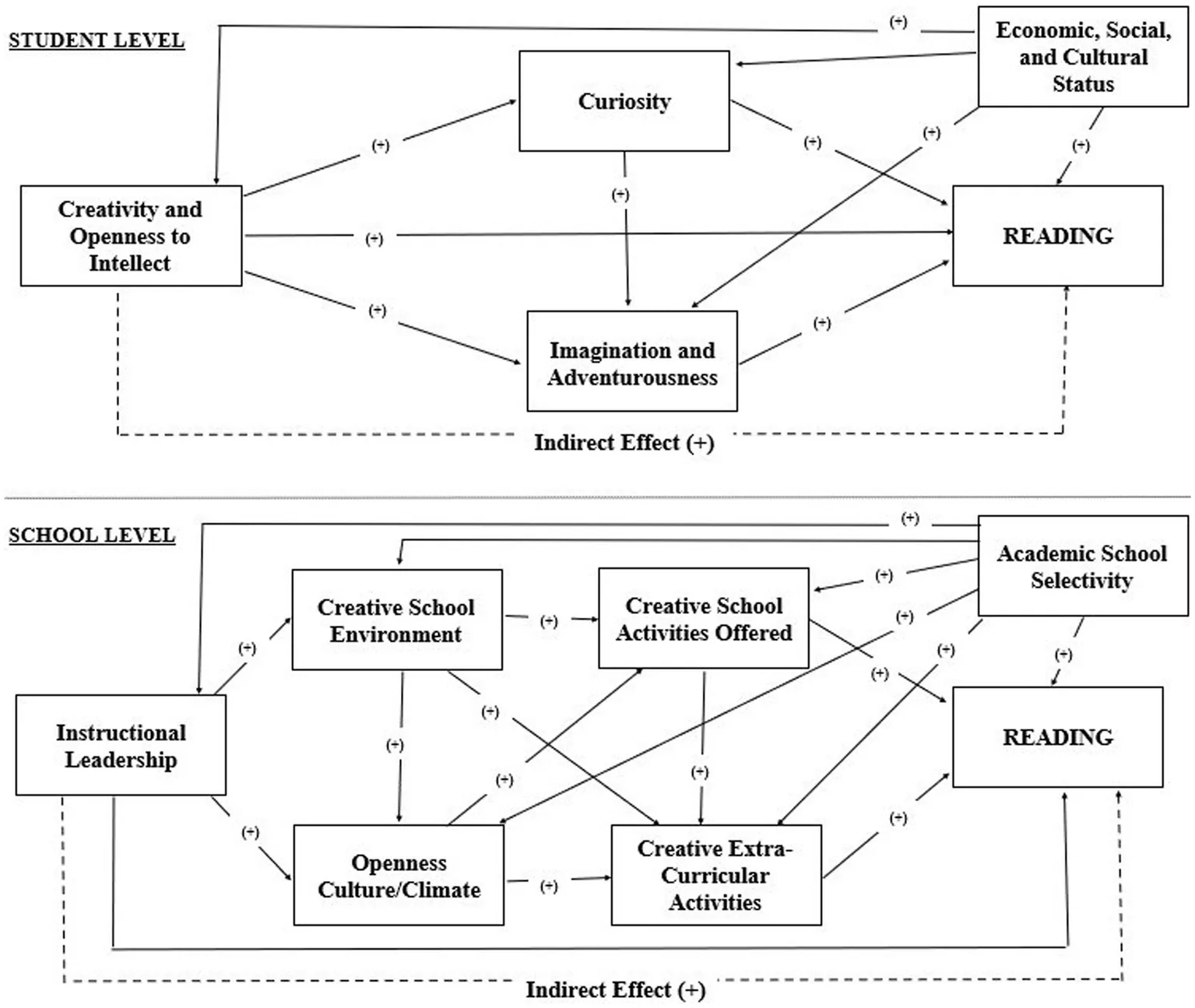
Conceptual framework. Source: Authors’ own creation.

## 4 The current study

This study aims to examine the influences of student-level creativity characteristics and school-level creativity-supportive conditions on reading performance using the PISA 2022 data for Türkiye within the framework of a multilevel path analysis and Bayesian estimation. More precisely, we investigated whether student curiosity (CURIOAGR) and imagination and adventurousness (IMAGINE) mediate the relationship between creativity and openness to intellect (CREATOP) and reading achievement at the student level. We also included students’ economic, social, and cultural status (ESCS) as a control variable at this level and examined its relationships with student-level variables. At the school level, we investigated whether creative school environment (CREENVSC), openness culture and climate (OPENCUL), creative school activities offered (ACTCRESC), and creative extra-curricular activities (CREACTIV) mediate the relationship between instructional leadership (INSTLEAD) and reading achievement (Figure 1). School selectivity (SCHSEL) was included as a control variable, and its relationships with school-level variables were examined. In this context, this study primarily addresses the following research questions:

RQ1. To what extent do creativity and openness to intellect (CREATOP) influence reading achievement at the student level, controlling for economic, social, and cultural status (ESCS)?

RQ2. Do curiosity (CURIOAGR) and imagination and adventurousness (IMAGINE) mediate the relationship between creativity and openness to intellect and reading achievement at the student level?

RQ3. To what extent does instructional leadership (INSTLEAD) influence reading achievement at the school level, controlling for academic school selectivity (SCHSEL)?

RQ4. Do creative school environment (CREENVSC), openness culture/climate (OPENCUL), creative school activities offered (ACTCRESC), and creative extra-curricular activities (CREACTIV) mediate the relationship between instructional leadership and reading achievement at the school level?

## 5 Methods

We used a cross-sectional research design drawing on the PISA 2022 student and school datasets. In such designs, data on dependent, mediator, and independent variables were gathered at a single time point through the same questionnaire. The following section outlines the study’s data sources, sample characteristics, measurement approach, and analytical strategy.

### 5.1 Data source and sample

This study relied on the Türkiye student and school questionnaire data from the 2022 Programme for International Student Assessment (PISA). A total of 7,250 fifteen-year-old students from Türkiye took part in PISA 2022. Of these participants, 49.1% (*n* = 3,561) were female, and 50.9% (*n* = 3,689) were male. In addition, 196 school principals from Türkiye participated. Among them, 87.2% (*n* = 171) were employed in public schools and 12.3% (*n* = 24) in private schools, with one principal not reporting the school type. The participating schools had an average enrollment of 696 students and an average teacher–student ratio of 13.76. On average, 36.99 students per school took part in the assessment. According to Hox et al. (2010), multilevel analyses require at least 50 groups and a minimum of five participants per group. Based on these criteria, the clustered PISA 2022 data structure was considered suitable for multilevel modeling.

OECD’s PISA sampling in Türkiye follows a stratified two-stage probability design implemented by the Ministry of National Education. The 2022 sampling frame covered all 12 NUTS-1 (Nomenclature of Territorial Units for Statistics, Level 1) statistical regions of the country: İstanbul, Batı Marmara, Ege, Doğu Marmara, Batı Anadolu, Akdeniz, Orta Anadolu, Batı Karadeniz, Doğu Karadeniz, Kuzeydoğu Anadolu, Ortadoğu Anadolu, and Güneydoğu Anadolu. Stratification variables included NUTS-1 region, school type (public/private, general/vocational), and school location (urban/rural), and schools were selected with probability proportional to size within each stratum (OECD, 2024a). The sample therefore reflects the full geographic and socio-economic distribution of 15-year-old students enrolled in Turkish secondary education, rather than a subset of regions. Because student and school weights are already calibrated to this population, all reported estimates could be generalized to the target population of 15-year-olds attending schools in Türkiye.

### 5.2 Variables and measures

This section outlines the variables and measurement tools employed in the study. All variables in the study were derived from the published PISA 2022 instruments. Student-level variables (PVs for reading, CREATOP, CURIOAGR, IMAGINE, and ESCS) came from the student questionnaire and cognitive assessment (item batteries listed below). School-level variables (INSTLEAD, CREENVSC, OPENCUL, ACTCRESC, CREACTIV, and SCHSEL) came from the school questionnaire administered to principals (OECD, 2024a).

The psychometric properties of these scales (including measurement invariance across participating countries, factor structure, and internal-consistency reliabilities) were evaluated separately for each country and language version by the OECD before data release. Detailed country-level reliability estimates for Türkiye, as well as evidence of scalar or partial scalar invariance across countries, are reported in the PISA 2022 Technical Report (OECD, 2024a). In addition, all questionnaires were adapted into Turkish following the TRAPD procedure (Translation, Review, Adjudication, Pretesting, and Documentation), which includes double translation, expert reconciliation, linguistic verification, and field-trial pretesting within the Turkish sample (OECD, 2024a).

Ten plausible values and OECD-developed indices were used as key measures. To construct these scales, the OECD applied the Rasch model for dichotomous items and the Partial Credit Model (PCM) for items with multiple response categories (OECD, 2023b). The Rasch model, a foundational technique within item response theory, is used to assess latent competencies or knowledge. It is particularly suitable for binary items (e.g., right/wrong or yes/no) and relies on a single difficulty parameter for each item. The model estimates both an individual’s underlying ability and the difficulty level of each item on a common scale, determining the probability of a correct response—this probability rises as a person’s ability surpasses the item’s difficulty. Key strengths of the Rasch model include ensuring comparability across different subgroups, facilitating the identification of item bias through differential item functioning, and producing sample-independent estimates, which enhance the validity and reliability of educational and psychological measurement (Wang and Wilson, 2005).

The Partial Credit Model, used in PISA 2022 to construct several indices, is another item response theory model designed for ordered, multi-category responses, such as Likert-type questionnaire items. PCM estimates respondents’ latent traits (e.g., dispositions or behaviors) and assigns multiple threshold parameters to items, each marking the difficulty of advancing from one response category to the next (for example, from “disagree” to “agree”). By extending the Rasch framework to polytomous data with varying thresholds, the PCM provides a more appropriate structure for the complex attitudinal and contextual scales included in PISA’s questionnaires for students, teachers, and schools (Wang and Wilson, 2005).

In OECD-generated indices such as those used in PISA, scores are standardized so that the OECD-wide mean equals 0 and the standard deviation equals 1. Positive scores represent values above the OECD average on the measured construct (e.g., socio-economic conditions, school selectivity, or curiosity), indicating relatively stronger or more favorable outcomes. Negative scores reflect values below the OECD average, suggesting comparatively lower or less favorable outcomes, though these values are not inherently negative. For example, a negative index score signals that students’ responses were less positive than the OECD mean, while a positive score indicates more positive responses overall (OECD, 2023b).

#### 5.2.1 Reading

The dependent variable for this study is students’ reading achievement, represented by the plausible values (PVs) provided in the PISA 2022 dataset. These PVs consist of ten estimated scores (PV1READ–PV10READ) that reflect each student’s underlying reading competence, derived through item response theory procedures. The OECD generates these values using the Rasch model for dichotomous items and extended models, such as the partial credit model, for items with multiple response categories. The PVs are scaled to an OECD mean of 500 and a standard deviation of 100 and are designed to incorporate both measurement error and imputation uncertainty. As such, they allow for robust multilevel analyses of reading outcomes while retaining the full variability of student performance (OECD, 2024a).

#### 5.2.2 Creativity and openness to intellect (CREATOP)

The independent variable of this study at the student level was creativity and openness to intellect. The CREATOP index was used to measure this variable. This index captured students’ tendencies to engage with abstract or complex information through reasoning and to interact with such material effectively. It reflects a personality dimension associated with creativity, encompassing students’ intellectual curiosity, openness to new ideas, and enjoyment of cognitively challenging tasks. In PISA 2022, this index was constructed based on the ST340Q01JA–ST340Q10JA items in the student questionnaire (OECD, 2024b).

#### 5.2.3 Curiosity (CURIOAGR)

One of the mediator variables of this study at the student level was curiosity. Curiosity was measured by using the CURIOAGR index in the PISA 2022 dataset. This index captured students’ inclination toward intellectual inquiry, learning, and exploration, and was conceptualized as a socio-emotional characteristic that supports creative thinking. Curiosity was measured by using the items covering openness to new ideas, persistence, and imagination, and contributes to students’ capacity for everyday innovation. This index was based on 10 items from the student questionnaire: ST301Q01JA–ST301Q10JA (OECD, 2024b).

#### 5.2.4 Imagination and adventurousness (IMAGINE)

One of the mediator variables of this study at the student level was imagination and adventurousness. We employed the IMAGINE index to measure this variable. Conceptualized as a socio-emotional characteristic that supports creative thinking, the index reflected students’ enjoyment of generating new ideas, their spontaneity, preference for avoiding routine, and willingness to explore new places. It was constructed by using items reflecting the divergent thinking component of creativity, abilities such as producing original ideas, forming flexible associations, breaking established patterns, experimenting with novel approaches, and considering problems from multiple perspectives. The index was based on seven items from the student questionnaire: ST342Q01JA–ST342Q08JA (OECD, 2024b).

#### 5.2.5 Instructional leadership (INSTLEAD)

The independent variable of this study at the school level was instructional leadership. We employed the INSTLEAD index to measure this variable. This index reflected actions such as providing teachers with feedback based on classroom observations, encouraging collaboration among teachers to develop new instructional approaches, and fostering a sense of responsibility among teachers for improving their instructional skills and enhancing student learning outcomes. The items used to measure this variable encompass leadership behaviors aimed at strengthening the quality of teaching, teacher motivation, and student achievement. It was constructed from five items from the school questionnaire: SC201Q01JA–SC201Q06JA (OECD, 2024a).

#### 5.2.6 Creative school environment (CREENVSC)

One of the mediator variables of this study at the school level was the creative school environment. We employed the CREENVSC index to measure this variable. This index reflected actions such as teachers’ assessment of students’ creativity, the extent to which classroom activities encourage students to devise new approaches for solving complex tasks, the promotion of risk-taking in learning, opportunities for students to express their ideas, collaboration on creative projects, and the creation of a supportive environment for sharing creative ideas. It was constructed from six items from the school questionnaire: SC205Q01JA–SC205Q07JA (OECD, 2024b).

#### 5.2.7 Openness culture/climate (OPENCUL)

One of the mediator variables of this study at the school level was openness culture/climate. This index reflected principals’ views on students’ inclination to be creative, their enjoyment of learning new things, and the value they place on cultural diversity. Items used to measure this variable covered inclusiveness, creativity, and tolerance for diversity among students. It was constructed from nine items from the school questionnaire: SC208Q01JA–SC208Q09JA (OECD, 2024b).

#### 5.2.8 Creative school activities offered (ACTCRESC)

One of the mediator variables of this study at the school level was creative school activities offered. We employed the ACTCRESC index to measure this variable. This index reflected the extent to which schools offer creative activities such as art, music, and drama, and was considered a component of a school environment that supports creative thinking. The index was constructed from eight items from the school questionnaire: SC207Q01JA–SC207Q08JA (OECD, 2024b).

#### 5.2.9 Creative extra-curricular activities (CREACTIV)

One of the mediator variables of this study at the school level was creative extra-curricular activities. We employed the CREACTIV index to measure this variable. This index reflected the structural supports within the school that foster creativity and captures school resources that indirectly contribute to the development of creative thinking skills. The index was constructed from three items from the school questionnaire: SC053Q01TA, SC053Q02TA, and SC053Q09TA (OECD, 2024b).

#### 5.2.10 Control variables

We included students’ economic, social, and cultural status (ESCS) as a control variable at the individual level. The ESCS index is constructed by standardizing and combining weighted information from several question groups in the PISA 2022 student questionnaire that capture parental education, parental occupational status, and household possessions (ST251, ST254, ST255, ST005, ST006, ST007, ST008, ST014, and ST015).

At the school level, we controlled for academic school selectivity (SCHSEL). The SCHSEL index in PISA 2022 indicates the extent to which schools rely on academic criteria when admitting students. It captures the frequency with which schools consider students’ prior academic achievement and recommendations from feeder schools, using a categorical response format. This index is derived from the SC012 item set in the school questionnaire.

### 5.3 Analytical strategy

This study made use of the OECD-generated indices and the ten plausible values for reading achievement. Each plausible value served as the dependent variable in separate analyses, and the results across these ten models were synthesized using Rubin’s pooling procedure. Rubin’s method—commonly applied in contexts involving multiply imputed data—combines estimates by averaging the parameter values obtained from each imputed dataset. It then incorporates both the average within-imputation variance and the between-imputation variability, along with a small correction factor to account for the uncertainty introduced through imputation. This approach ensures that statistical inferences reflect both the observed data and the uncertainty surrounding imputed values, thereby producing more accurate and defensible conclusions (Jewsbury et al., 2024).

All statistical analyses were conducted using Mplus Version 8.3. Missing data were addressed through Full Information Maximum Likelihood (FIML) paired with robust maximum likelihood estimation (MLR), allowing for consistent estimates even under conditions of non-normality. FIML yields unbiased parameters when the missingness mechanism is missing at random (MAR) (Muthén and Muthén, 2017). The analysis proceeded in three stages. The first stage involved preliminary descriptive examinations, including the computation of arithmetic means, standard deviations, intra-class correlations (ICCs), and Pearson correlations. At this point, mean values, standard deviations, and ICCs were pooled across the ten plausible values for reading.

In the second stage, multilevel path analysis was performed. To account for the complex sampling design, the final student weight (FSTUWT) was applied using the WEIGHT = FSTUWT and CLUSTER = CNTSCHID commands in Mplus. Model evaluation included the reporting of free parameters, chi-square statistics (χ^2^), degrees of freedom (df), chi-square significance values, the MLR scaling correction factor, and global fit indices such as the Root Mean Square Error of Approximation (RMSEA), Comparative Fit Index (CFI), Tucker–Lewis Index (TLI), and the within- and between-level Standardized Root Mean Square Residual (SRMR). Following recommendations for large-sample models, acceptable fit was defined as χ^2^/df ≤ 5, RMSEA ≤ 0.07, SRMR ≤ 0.08, and CFI and TLI ≥ 0.92 (Hair et al., 2014). Standardized and unstandardized coefficients, standard errors, and *p*-values were reported, and pooled estimates were calculated for all model parameters and fit indices.

The third stage employed Bayesian estimation to assess indirect effects. Bayesian methods yield full posterior distributions, enabling more reliable credible intervals for indirect effects and better quantification of parameter uncertainty compared to frequentist path analysis. Moreover, they offer advantages for multilevel mediation models by producing full posterior distributions and accommodating complex hierarchical structures. Indirect effects were calculated as the product of the relevant path coefficients within multilevel mediation frameworks, with parameters estimated using Markov Chain Monte Carlo (MCMC) procedures. Non-informative priors were used for regression paths and variance components to ensure model flexibility and suitability for nested student–school data. The statistical significance of indirect effects was determined by evaluating whether the 95% credible intervals excluded zero, allowing for probability-based interpretations of mediation processes (Miočević et al., 2018).

All structural regression (path) coefficients, including the within-level paths and the between-level paths (and the corresponding leadership/climate paths feeding the indirect effects), were assigned an improper flat prior, N(0, ∞), placing no constraint on the plausible range of these coefficients. All residual (disturbance) variances, at both the within level and the between level, were assigned an inverse-gamma prior, IG(−1, 0), which is Mplus’s standard non-informative (improper) default for variance parameters. We adopted these Mplus default settings rather than specifying custom informative priors because no external, study-specific prior information (e.g., from prior applications of this exact model and population) was available to justify alternative hyperparameter values. Using the software’s non-informative defaults therefore allowed the observed data, rather than subjective prior belief, to dominate the posterior distributions, which is standard practice in applied Bayesian SEM when no strong prior evidence exists. This specification is consistent with van de Schoot et al.'s (2021) recommendation that Mplus’s standard non-informative priors are appropriate when no reliable prior information exists, as they allow the data to dominate posterior inference without imposing potentially misleading constraints. They further note that researchers may use such priors for exploratory purposes and let the data predominantly shape the posterior when there is a lack of certainty regarding the model parameters (van de Schoot et al., 2021).

Moreover, considering the sample size of this study (*N* = 7,250 individuals nested within 196 clusters, yielding an average cluster size of 36.99), the likelihood function was expected to dominate the prior distributions regardless of their diffuseness. The number of clusters exceeds common recommendations for stable estimation of between-level parameters in multilevel SEM (i.e., 50 or more clusters) (Hox et al., 2010), and the large within-level sample provides substantial information for the within-level structural paths. Under such conditions, the influence of non-informative or flat priors on the posterior distribution asymptotically diminishes as sample size increases (Gelman et al., 2013), making the choice between Mplus’s default priors and mildly informative alternatives unlikely to meaningfully alter the substantive conclusions. This is consistent with van de Schoot et al.'s (2021) observation that with sufficiently large and well-structured data, non-informative priors allow the likelihood to predominantly shape the posterior, reducing sensitivity to the specific hyperparameters chosen. To further substantiate this choice, a sensitivity analysis was conducted in which the default inverse-gamma prior for all residual (disturbance) variances, IG(−1, 0), was replaced with an alternative, commonly used proper non-informative specification, IG(0.001, 0.001), at both the within and between levels, while all other model specifications were held constant. The standardized (STDYX) structural coefficients, including the direct, indirect, and total effects reported in the main analysis, remained unchanged to the second decimal place under this alternative prior specification. This invariance indicates that the substantive conclusions of the model are not sensitive to the particular choice of non-informative prior for the variance parameters, and confirms that the likelihood dominates the posterior distribution regardless of which non-informative prior is adopted for these parameters.

Beyond the general merits of Bayesian estimation, three features of the present model make the Bayesian framework particularly well suited to our data. First, the number of Level-2 units (schools) was small to moderate, and maximum-likelihood-based two-level SEM is known to produce inadmissible solutions (Heywood cases or negative variance estimates for random intercepts and slopes) when the Level-2 sample size is limited. The Bayesian approach, through its priors on variance components, keeps these parameters within the admissible (positive-definite) parameter space (Hox et al., 2010; McNeish and Stapleton, 2016). Second, the model includes multiple indirect effects. The classical Sobel test relies on the assumption that the product of coefficients is normally distributed, and bootstrap-based tests can be unstable in small samples; the Bayesian approach, by contrast, yields the full posterior distribution of each indirect effect and provides 95% credible intervals that are allowed to be asymmetric—an important property given the inherently non-normal distribution of product terms (Miočević et al., 2018). Third, the model combines a complex two-level structure with plausible values from large-scale assessment data (PISA), a configuration that ML-based two-level SEM frequently encounters convergence problems with. MCMC estimation has been shown to be considerably more stable in such complex models (Muthén and Asparouhov, 2012; van de Schoot et al., 2014).

For the Bayesian models, pooled values for the number of free parameters, the observed and replicated chi-square statistics, and the posterior predictive *p*-value (PPP) were reported as indicators of model fit. A PPP around 0.50, together with substantial overlap between the observed and replicated chi-square distributions (i.e., a difference interval containing zero), is commonly interpreted as evidence of adequate model fit, whereas values close to 0 or 1 indicate poor fit (Gelman and Rubin, 1992). Finally, pooled unstandardized estimates, posterior standard deviations, one-tailed *p*-values, and 95% credible interval bounds were presented for the total, direct, and indirect effects.

## 6 Results

This section outlines the findings of the study, organized into three parts: preliminary analyses, structural equation modeling outcomes, and Bayesian estimation results for the indirect pathways.

### 6.1 Preliminary analysis

This part reports the descriptive statistics, including means, standard deviations, ICCs, and Pearson correlations. The results of these initial analyses are summarized in Table 1.

**Table 1:** Means, standard deviations, ICCs, and Pearson correlation coefficients.

Variables	M	SD	ICC	α	1	2	3	4	5	6	7	8	9	10
Dependent V.														
1. Reading	451.163^†^	87.225^†^	0.508^†^	-	-									
Student-level														
2. CREATOP	0.118	1.069	0.031	0.810	178***	-								
3. CURIOAGR	0.332	1.147	0.035	0.700	209***	0.391***	-							
4. IMAGINE	0.252	1.110	0.055	0.680	269***	0.436***	0.317***	-						
5. ESCS	−1.270	1.164	0.344	-	0.337***	0.118***	0.067***	0.105***	-					
School-level														
6. INSTLEAD	0.570	1.011	-	0.820	−0.133***	−0.017	−0.010	−0.017	−0.049***	-				
7. CREENVSC	0.346	1.079	-	0.900	0.106***	0.048***	0.064***	0.049***	0.090***	0.263***	-			
8. OPENCUL	0.049	1.063	-	0.910	0.168***	0.052***	0.055***	0.047***	0.130***	0.186***	0.498***	-		
9. ACTCRESC	0.011	0.875	-	0.810	0.115***	0.017	0.027*	0.013	0.127***	0.191***	0.110***	0.134***	-	
10. CREACTIV	1.91	1.014	-	-	0.165***	0.030*	0.029*	0.033**	0.179***	0.113***	0.133***	0.286***	0.354***	-
11. SCHSEL	2.26	0.846	-	-	0.297***	0.043***	0.055***	0.055***	0.178***	−0.090***	0.150***	0.235***	0.079***	0.025*

† = Pooled values; *p < 0.05; **p < 0.01; ***p < 0.001; CREATOP, Creativity and openness to intellect; CURIOAGR, Curiosity; IMAGINE, Imagination and adventurousness; ESCS, index of economic, social, and cultural status; INSTLEAD, Instructional leadership; CREENVSC, Creative school environment; OPENCUL, Openness culture/climate; ACTCRESC, Creative school activities offered; CREACTIV, Creative extra-curricular activities; SCHSEL, index of academic school selectivity.

The descriptive statistics, Cronbach’s Alpha coefficients, and bivariate correlations for all study variables are presented in Table 1. Internal consistency was evaluated using Cronbach’s alpha coefficients provided by the OECD (2024a) for Turkish sample. Reliability estimates ranged from 0.68 to 0.91. Most scales showed good to excellent internal consistency (α = 0.81–0.91), while curiosity (CURIOAGR) met the conventional 0.70 threshold for acceptable reliability. Imagination/adventurousness (IMAGINE) (α = 0.68) fell slightly below this criterion, indicating that results related to this scale should be interpreted with caution.

Reading performance showed moderate between-school variation, as reflected in a pooled ICC of 0.508. At the student level, all three creativity-related dispositions—creativity/openness to intellect (CREATOP), curiosity (CURIOAGR), and imagination/adventurousness (IMAGINE)—were positively associated with reading performance (r values ranging from 0.178 to 0.269, *p* < 0.001). Student socioeconomic status (ESCS) was also positively correlated with reading scores (r = 0.337, *p* < 0.001).

At the school level, several contextual variables demonstrated small but significant associations with reading outcomes. Instructional leadership (INSTLEAD) correlated negatively with reading (r = −0.133, *p* < 0.001). In contrast, a creative school environment (CREENVSC), openness culture (OPENCUL), creative school activities (ACTCRESC), and creative extracurricular opportunities (CREACTIV) were all positively related to reading performance (rs = 0.106 to 0.168, all *p* < 0.001). Academic school selectivity (SCHSEL) showed the strongest school-level association with reading (r = 0.297, *p* < 0.001).

Correlations among the predictors reveal meaningful clustering. Student-level creativity dispositions were moderately interrelated (rs = 0.317 to 0.436), and ESCS showed small positive associations with all three. At the school level, creative environment, openness culture, and creative activity indicators were moderately intercorrelated (rs = 0.110 to 0.498), suggesting a coherent creative-climate dimension across schools.

Overall, the pattern of correlations indicates that both student-level dispositions related to creativity and school-level features that support creativity are positively correlated with reading achievement. These relationships justify the inclusion of both individual and contextual variables in the subsequent multilevel models.

### 6.2 Path analysis results

We performed 10 path analyses to examine the model fit and direct relationships between study variables, using each plausible value of reading as the dependent variable, and then pooled the results using Rubin’s pooling method. The pooled model fit indices revealed an acceptable fit to the data: number of free parameters = 46; chi-square value = 9.521; degrees of freedom = 4; *p*-value = 0.049; scaling correction factor for MLR = 0.883; RMSEA = 0.014; CFI = 0.998; TLI = 0.986; SRMR (within) = 0.000; SRMR (between) = 0.037. Table 2 shows the pooled path analysis results regarding student- and school-level analysis.

**Table 2:** Path analysis results (pooled).

Paths	B	S.E.	β (standardized)	S.E. (standardized)	*p*
Student-level					
CREATOP → Reading	−0.066	0.983	0.001	0.017	0.947
CURIOAGR → Reading	5.433	1.039	0.102	0.019	0.000***
IMAGINE → Reading	8.207	0.900	0.148	0.016	0.000***
CREATOP → CURIOAGR	0.426	0.017	0.395	0.014	0.000***
CREATOP → IMAGINE	0.375	0.016	0.361	0.015	0.000***
CURIOAGR → IMAGINE	0.171	0.014	0.178	0.014	0.000***
ESCS → Reading	3.778	0.951	0.072	0.018	0.000***
ESCS → CREATOP	0.108	0.013	0.118	0.014	0.000***
ESCS → CURIOAGR	0.016	0.011	0.016	0.011	0.146
ESCS → IMAGINE	0.049	0.011	0.052	0.012	0.000***
School-level					
INSTLEAD → Reading	−10.722	4.036	−0.184	0.069	0.008**
ACTCRESC → Reading	5.206	4.092	0.078	0.069	0.203
CREACTIV → Reading	11.657	3.883	0.208	0.068	0.003**
INSTLEAD → CREENVSC	0.274	0.088	0.251	0.079	0.002**
INSTLEAD → OPENCUL	0.099	0.072	0.093	0.069	0.166
CREENVSC → OPENCUL	0.428	0.072	0.435	0.058	0.000***
CREENVSC → ACTCRESC	0.044	0.065	0.055	0.083	0.503
CREENVSC → CREACTIV	−0.027	0.063	−0.028	0.066	0.669
OPENCUL → ACTCRESC	0.101	0.076	0.124	0.094	0.183
OPENCUL → CREACTIV	0.267	0.070	0.276	0.070	0.000***
ACTCRESC → CREACTIV	0.385	0.085	0.324	0.066	0.000***
SCHSEL → Reading	25.738	4.403	0.375	0.059	0.000***
SCHSEL → INSTLEAD	−0.082	0.086	−0.070	0.072	0.335
SCHSEL → CREENVSC	0.258	0.089	0.200	0.068	0.004**
SCHSEL → OPENCUL	0.220	0.074	0.174	0.056	0.003**
SCHSEL → ACTCRESC	0.053	0.074	0.052	0.067	0.444
SCHSEL → CREACTIV	−0.075	0.076	−0.061	0.062	0.324

*p < 0.05; **p < 0.01; ***p < 0.001; CREATOP, Creativity and openness to intellect; CURIOAGR, Curiosity; IMAGINE, Imagination and adventurousness; ESCS, index of economic, social, and cultural status; INSTLEAD, Instructional leadership; CREENVSC, Creative school environment; OPENCUL, Openness culture/climate; ACTCRESC, Creative school activities offered; CREACTIV, Creative extra-curricular activities; SCHSEL, index of academic school selectivity.

The multilevel path analysis results in Table 2 revealed several notable patterns. At the student level, curiosity and imagination showed substantive positive associations with reading performance, both in raw and standardized terms. Imagination had the strongest relationship with reading performance (*β* = 0.148, *p* < 0.001), followed by curiosity (*β* = 0.102, *p* < 0.001). Creativity/openness to intellect (CREATOP) was not directly related to reading, but it had strong relationships with both curiosity (β = 0.395, *p* < 0.001) and imagination (β = 0.361, *p* < 0.001), suggesting its influence on achievement operates indirectly through these dispositions. ESCS had a modest direct relationship with reading (β = 0.072, *p* < 0.001) and was also positively related to CREATOP and IMAGINE, indicating that socioeconomic background shapes both cognitive-motivational traits and performance. The only non-significant student-level path was ESCS → CURIOAGR.

At the school level, several institutional variables were associated with reading outcomes. Creative extra-curricular activities (CREACTIV) were positively related to reading (*β* = 0.208, *p* = 0.003), whereas creative activities offered within the classroom (ACTCRESC) were not. Instructional leadership was negatively associated with reading (*β* = −0.184, *p* = 0.008). School selectivity (SCHSEL) was the strongest school-level predictor of reading (*β* = 0.375, *p* < 0.001), consistent with stratification effects.

The structural relations among school-level constructs also revealed a coherent pattern. Instructional leadership had a direct relationship with the creative school environment (*β* = 0.251, *p* = 0.002), which in turn was strongly related to openness culture (*β* = 0.435, *p* < 0.001). Openness culture was positively associated with CREACTIV (*β* = 0.276, *p* < 0.001), as was ACTCRESC (*β* = 0.324, *p* < 0.001), suggesting that a supportive and open school climate fosters a richer creative activity profile. School selectivity was significantly related to multiple cultural and environmental features, including the creative environment (*β* = 0.200, *p* = 0.004) and openness culture (*β* = 0.174, *p* = 0.003), though it did not directly shape creative activity offerings.

To examine whether the inclusion of mediator variables suppressed the relationship between INSTLEAD and reading, we estimated an alternative path analysis model in which INSTLEAD was the sole independent variable at the school level. Pooled results revealed a negative association between instructional leadership and reading (*β* = −0.160, SE = 0.073, *p* < 0.05). Given that this coefficient was slightly smaller in magnitude than that obtained in the full model (*β* = −0.184), the suppression effect appears to be minimal.

Taken together, the findings indicated that student dispositions related to curiosity and imagination were key drivers of reading performance, largely shaped by broader cognitive-personality traits and socioeconomic conditions. At the school level, creative school environments and extra-curricular activities contributed meaningfully to achievement, while academic selectivity remained a dominant structural factor. The pattern of significant pathways suggested a model in which both individual dispositions and school climates contributed to literacy outcomes, but through partially distinct mechanisms.

### 6.3 Bayesian estimation results

We performed Bayesian estimation to test indirect effects. Posterior distributions were estimated using Mplus’s Gibbs sampler with four independent MCMC chains. A minimum of 15,000 iterations per chain was requested for each of the ten analyses, with the first half discarded as burn-in. Convergence was assessed using the Gelman–Rubin Potential Scale Reduction (PSR) statistic, adopting a stringent criterion of PSR < 1.05 for all parameters (Gelman and Rubin, 1992). The diagnostics indicated that PSR values stabilized around 1.001 well before the 2,000th iteration and remained below the 1.05 threshold for every parameter, providing clear evidence of successful convergence. Pooled model fit was subsequently evaluated. Consistent with the path analysis results, the pooled fit indices from Bayesian estimation suggested an acceptable fit to the data: number of free parameters = 46; the 95% confidence interval for the difference between the observed and replicated chi-square values ranged from −26.786 to 30.733, containing zero; posterior predictive *p*-value (PPP) = 0.414 (Table 3).

**Table 3:** Bayesian estimation results regarding direct and indirect effects (pooled).

Paths	B	Posterior SD	Lower CI	Upper CI	*p*
Student-level					
CREATOP → Reading (Direct Effect)	−0.071	0.978	−1.987	1.845	0.471
CREATOP → CURIOAGR → Reading	2.318	0.413	0.1.508	3.128	0.000***
CREATOP → IMAGINE → Reading	3.064	0.350	2.378	3.749	0.000***
CREATOP → CURIOAGR → IMAGINE → Reading	0.593	0.078	0.439	0.747	0.000***
Total indirect effects	5.978	0.496	5.006	6.950	0.000***
School-level					
INSTLEAD → Reading (direct effect)	10.643	3.903	−18.293	−2.994	0.003**
INSTLEAD → CREENVSC → ACTCRESC → Reading	0.031	0.147	−0.257	0.319	0.834
INSTLEAD → OPENCUL → CREACTIV → Reading	0.259	0.274	−0.278	0.796	0.344
INSTLEAD → CREENVSC → CREACTIV → Reading	−0.067	0.256	−0.568	0.434	0.794
INSTLEAD → OPENCUL → ACTCRESC → Reading	0.026	0.090	−0.150	0.202	0.772
INSTLEAD → CREENVSC → OPENCUL → ACTCRESC → Reading	0.042	0.086	−0.126	0.211	0.624
INSTLEAD → CREENVSC → OPENCUL → ACTCRESC → CREACTIV → Reading	0.043	0.050	−0.055	0.141	0.390
INSTLEAD → CREENVSC → OPENCUL → CREACTIV → Reading	0.328	0.206	−0.077	0.732	0.112
Total indirect effects	0.759	0.460	−0.143	1.660	0.100

*p < 0.05; **p < 0.01; ***p < 0.001; CREATOP, Creativity and openness to intellect; CURIOAGR, Curiosity; IMAGINE, Imagination and adventurousness; INSTLEAD, Instructional leadership; CREENVSC, Creative school environment; OPENCUL, Openness culture/climate; ACTCRESC, Creative school activities offered; CREACTIV, Creative extra-curricular activities.

The Bayesian estimation of direct and indirect effects in Table 2 provided further insight into the mechanisms through which individual and school-level variables shape reading performance. Direct effects of Bayesian estimation results at both the student and school levels supported those of the path analysis results, which implies the robustness of the path analysis results. At the student level, all indirect pathways from creativity/openness to intellect (CREATOP) to reading were statistically significant and positive. The largest specific indirect effect operated through imagination (B = 3.064, 95% CI [2.378, 3.749], *p* < 0.001), followed by the pathway through curiosity (B = 2.318, 95% CI [1.508, 3.128], *p* < 0.001). A third, sequential route—CREATOP → CURIOAGR → IMAGINE → reading—also contributed meaningfully (B = 0.593, 95% CI [0.439, 0.747], *p* < 0.001). The total indirect effect was substantial (B = 5.978, 95% CI [5.006, 6.950], *p* < 0.001), confirming that the influence of CREATOP on reading was fully mediated by these motivational and imaginative dispositions. This strengthens the interpretation that creativity-related traits support reading achievement primarily through fostering curiosity and imaginative engagement.

In contrast, none of the school-level indirect effects of instructional leadership on reading reached statistical significance. Across all pathways—whether mediated through creative school environments, openness culture, curricular creative activities, or extracurricular creative opportunities—the confidence intervals included zero, and *p*-values are non-significant. Even the longest chain models (e.g., instructional leadership → creative environment → openness culture → activity offerings → reading) yield small and statistically uncertain effects. The total school-level indirect effect (B = 0.759, 95% CI [−0.143, 1.660], *p* = 0.100) similarly indicated no reliable mediated influence.

These findings suggested that while school leadership might shape aspects of school climate and creative activity structures, these institutional features did not translate into measurable indirect effects on reading performance within this model. Together, the results highlighted a strong and coherent mediational structure at the student level—where creativity-related personality traits affected achievement through curiosity and imagination—contrasted with a weak or non-existent indirect influence of leadership-driven school processes on academic outcomes.

## 7 Discussion and conclusion

This study examined the influences of student-level creativity characteristics (creativity and openness to intellect, curiosity, and imagination and adventurousness) together with school-level educational leadership and creativity-supportive conditions (creative school environment, openness culture and climate, creative school activities offered, and creative extra-curricular activities) on reading performance using the PISA 2022 data for Türkiye within the framework of a multilevel path analysis.

The results indicate that at the student level, the effect of creativity and openness to intellect on reading performance is mediated by curiosity and imagination. At the school level, in contrast, none of the indirect effects of instructional leadership on reading performance—through a creative school environment, a culture of openness, or creative activities—were found to be significant.

### 7.1 Student-level results

Our most striking finding at the student level is that, although the direct effect of creativity and intellectual openness on reading performance was found to be nonsignificant, the indirect effect of this trait through curiosity and imagination was significant. Creativity motivation may stimulate curiosity and the desire to imagine in individuals. Curiosity, imagination, and adventurousness may, in turn, increase the desire to learn new information and interact with different stimuli, leading to more frequent reading, thereby enhancing reading performance.

The mediating role of curiosity in reading performance supports the theoretical claim that curiosity is an intrinsic source of motivation that guides information-seeking behavior and attentional processes (Markey and Loewenstein, 2014; Zhang et al., 2026). Similarly, Prasad and Hommel (2024) demonstrated in their study investigating how curiosity affects attentional control that high uncertainty conditions elicit a state of curiosity, and that this state enhances attentional processing of task-irrelevant stimuli. The results support the claim that curiosity is an intrinsic source of motivation guiding information-seeking processes. At the same time, this finding is consistent with the results of Gurning and Siregar (2017), which show that students with a high level of curiosity have higher reading comprehension achievement. Likewise, Singh and Manjaly (2022) demonstrate that curiosity could enhance academic achievement. Gurning and Siregar (2017) explain the effect of curiosity on academic achievement by emphasizing that highly curious students are more active in the learning process and make greater efforts to achieve the maximum result in reading comprehension. Students show higher comprehension and retention when reading high-interest passages and perform better academic outcomes (Markey and Loewenstein, 2014). Mussel (2022) found that curiosity is still one of the significant predictors of academic achievement, even if information-seeking behavior does not have a significant role in the relationship between curiosity and academic performance (Mussel, 2022).

The fact that imagination emerges as the strongest mediator at the student level aligns with Mar and Oatley’s (2008) view that reading is not merely a process of decoding, but a cognitive simulation process requiring the mental reconstruction of the world presented in the text. Similarly, Baki (2025) argues that creative reading is not merely constructing surface-level meaning, but goes beyond this to require discovering different meanings within the text and reconstructing the text itself. Indeed, instructions or exercises aimed at forming mental images during reading facilitate better comprehension of the text and may support semantic recall rather than verbatim memorization (Zwaan, 2016).

The finding that creativity and intellectual openness were not significantly related to reading performance suggests that these traits alone do not translate into performance. However, when motivational processes such as curiosity and imagination are activated, they enhance reading performance. This is further supported by the finding that the sequential pathway from creativity and intellectual openness to reading performance through curiosity and imagination is significant. This result is also similar to Meyer et al.’s (2023) study, in which intrinsic value was tested as a mediator in the relationship between openness to intellectual and language achievement, and intrinsic value was found to play a significant mediating role in this relationship. In this context, it can be argued that these three variables at the student level together emphasize that a motivational–cognitive system operates in affecting reading performance.

The role of socioeconomic and cultural status (ESCS), which we included in the model as a control variable, also points to a notable mechanism regarding reading performance. ESCS was directly and significantly related to reading performance. At the same time, it was significantly related to creativity, intellectual openness, and imagination. However, the fact that the direct effect of ESCS on curiosity was found to be insignificant is a noteworthy finding. This result suggests that socioeconomic resources do not shape epistemic orientation—at least not through a direct pathway — and supports the interpretation that curiosity is a relatively autonomous motivational source. While creativity, intellectual openness, and imagination are associated with socioeconomic status, curiosity seems to function as a relatively egalitarian internal source of motivation that is not shaped by socioeconomic advantage.

Our findings at the student level are consistent with international studies showing that socioeconomic and cultural advantage is transmitted to reading partly through psychological-motivational variables. Michael and Kyriakides (2023), in their study examining 38 European countries using PISA 2018 data, revealed that SES predicts reading achievement both directly and through motivational mechanisms. Similarly, Yeung et al. (2022), in their study using PISA 2018 data, showed that at the student level, SES positively predicts reading achievement through expectation and value, drawing attention to how more advantaged students feel greater self-efficacy and enjoy reading more. In a similar vein, Karaman (2022), in his study based on 2018 PISA Turkey data, found that ESCS is an effective determinant of students’ and schools’ reading achievement. The fact that similar results emerged in our study, which used 2022 PISA Turkey data, emphasizes the importance of the effect of ESCS on reading performance over the years.

A notable finding in the present study is that despite the insignificance of the direct effect, the strong total indirect effect of the creativity-openness trait on reading can be interpreted as full mediation. This mechanism is also consistent with studies suggesting that the effect of openness to experience on academic skills develops through intrinsic motivational and epistemic processes (e.g., Li et al., 2023). Li et al. (2023) state that individuals with higher levels of openness to experience are more motivated to learn and explore (intrinsic motivational processes) and tend to collect more diverse information (epistemic processes). This pattern is also corroborated by the following findings of von Stumm et al. (2011) that effort and intellectual curiosity are direct, correlated predictors of academic performance, indicating that a “hungry mind” is a core determinant of individual differences in academic achievement. Considering the exam-centered structure of the Turkish education system, the relationship between motivational mechanisms and achievement may indicate that intrinsic motivation remains a persistent resource even in high-stakes, stress-inducing education systems (e.g., Uzun et al., 2024).

### 7.2 School-level results

Unlike the student-level findings, the pattern at the school-level presented a notably different picture. Some studies on leadership (e.g., Leithwood et al., 2020) suggest that school leadership produces positive effects on student achievement. However, our school-level findings revealed that, contrary to our expectations, instructional leadership showed a negative direct relationship with reading performance. Three non-exclusive explanations warrant serious consideration. The first explanation involves reverse-causal or compensatory dynamics. Principals in low-performing schools may increase instructional interventions to boost success, which can make the leadership–achievement relationship appear reversed in cross-sectional data. This is because cross-sectional studies cannot measure the impact of leadership over time (Hallinger, 2011). Furthermore, the cross-sectional data may have been collected during a period when the leader was exerting intensive effort, but these efforts had not yet been reflected in student outcomes. Since the leader intervenes while the school’s reading performance is already low, it may seem as though causality is operating in reverse. In reality, the leader does not cause failure; rather, the leader intervenes because the school’s performance is low. Therefore, this situation indicates that, in cross-sectional PISA data, leadership is shaped internally within the school context rather than causally. That is, the leader’s behavior is shaped according to the school’s conditions; the school shapes leadership.

The second explanation concerns structural incongruence with the assessment regime. In systems dominated by high-stakes centralized examinations, the instructional leadership behaviors captured by PISA’s INSTLEAD scale (feedback derived from classroom observation, collaborative pedagogical improvement, and a teacher-development orientation) may be crowded out by the demands of examination preparation. Where such behaviors do persist, they may be perceived as competing with the exam-focused activities that principals, teachers, and families prioritize, rather than as complementary to them (Wheldon and Dobson, 2024). Under these conditions, a negative direct association between instructional leadership and achievement need not indicate that leadership is ineffective, but rather that its emphasis is misaligned with the outcomes the system most heavily rewards.

The third explanation concerns reporting inflation in higher-selectivity schools. In highly selective schools, principals may report lower instructional leadership scores, potentially because they may perceive less need for active instructional leadership in settings that already select for high prior achievement. The negative correlation between school selectivity and instructional leadership in our data (*r* = −0.09, *p* < 0.001) is consistent with this dynamic. Notably, the dominant coefficient at the school level—the direct path from selectivity to reading achievement (*β* = 0.375)—indicates that exam-based stratification accounts for most of the structural variation in outcomes. The role of instructional leadership is therefore best interpreted against this backdrop, as a secondary factor operating within a system whose achievement differences are shaped primarily by selective sorting (DiMartino and Jessen, 2016).

The strong direct effect of academic selectivity on reading reveals that exam-focused school stratification in Turkey is the dominant structural factor determining achievement and indicates that the impact of leadership should be interpreted within this structural inequality. In the Turkish education system, differences in selectivity between private and public schools or between schools that admit students through exams and those that do not may be the main factors fueling this mechanism. Indeed, in a large-scale study conducted by Suna et al. (2020) with data from two million students within the scope of Turkey’s high school placement system, it was found that students attending private schools—which represent a more socioeconomically advantaged school type—demonstrated significantly higher performance in language, mathematics, and science tests compared to other students.

The fact that the indirect paths from leadership to reading performance through creative school processes are not significant also points to another issue that requires examination. This result can be interpreted as a structural disconnect between the leadership processes shaping the school climate that highlight creativity and the academic processes determining reading in the Turkish education system.

Theoretical and empirical studies in the relevant literature predominantly posit that school principals influence student achievement primarily by fostering a conducive school climate—for example, by encouraging teachers’ professional development, building collaborative relationships, and setting high-performance expectations. Leadership practices are responsive to the school climate, whose components may vary depending on the institutional, political, and cultural contexts of different school systems (Zhu et al., 2026). However, as the present study suggests, school climate components aimed at nurturing student creativity and distinctive student outcomes may not mediate the relationship between school leadership and standardized student outcomes such as achievement on the PISA assessment. In other words, the indirect effects identified in this study should not be generalized to school climate components that emphasize standardized student outcomes in high-stakes examinations (Leithwood et al., 2020).

Moreover, the pressure of centralized exams may be inhibiting or reducing the impact of creative activities and a culture of openness on students’ academic performance. Indeed, high-stakes tests narrow pedagogical breadth and creativity, replacing them with instructional approaches focused on exam preparation and repetition-based learning (Wheldon and Dobson, 2024). At the same time, the insignificance of the indirect effect of creative activities on reading performance suggests that these activities may not have been designed in ways that develop reading skills. The positive and significant relationship between creative extracurricular activities (CREACTIV) and reading performance, on the other hand, suggests that voluntary and interest-based activities strengthen reading habits more than in-school official creativity programs. In fact, this again points to the importance of intrinsic motivational processes.

Overall, this study has shown that the reading performance of Turkish students is shaped not directly by individual personality traits such as creativity–openness, but through motivational-cognitive mechanisms such as curiosity and imagination. At the school level, the insignificance of the indirect effects of instructional leadership on reading through creative institutional processes indicates that there is a need to build a bridge between institutional creativity conditions and academic performance. These findings require that interventions aimed at improving reading should be designed not only around teaching cognitive skills but also around learning environments that spark curiosity and nurture imagination.

### 7.3 Theoretical and practical implications

The findings of the study have important implications not only for the creativity and academic achievement literature but also for education policy and school practice. In terms of theoretical contribution, firstly, the fact that the effect of the creativity–openness trait on reading performance is fully mediated reinforces the view that personality traits shape academic outcomes not directly, but through motivational–cognitive intermediaries. Secondly, by modeling curiosity and imagination as separate mediators, the study has empirically demonstrated that, although these two constructs are often used interchangeably in the literature, they provide distinct contributions to reading. Finally, as one of the first Turkish studies to use the creativity module of PISA 2022, it reveals the potential of this data source to serve as a reference for international comparative research.

From a practical perspective, the strong mediating role of curiosity and imagination on reading indicates that reading instruction should be redesigned not only as the acquisition of cognitive skills but within a framework of text selection and activity design that sparks curiosity and stimulates imagination. The fact that creative extracurricular activities directly predict reading demonstrates that voluntary activities such as after-school clubs, literature workshops, and reading communities should be regarded not as a burden, but as important practices for reading.

Although instructional leadership shapes school climate, its lack of reflection on reading suggests that leadership initiatives do not yield the expected outcome when not directly aligned with the design of creative activities; this necessitates that school administrators take an active role not only in academic supervision but also in the design and preparation of creative instructional environments. The prominence of academic selectivity as a predictor points to another issue: that the inequalities in reading performance are structurally rooted, and the fact that curiosity is found to be independent of socioeconomic background suggests that curiosity-driven reading practices could serve as an equalizing tool for disadvantaged students.

### 7.4 Limitations and future implications

Our study, in addition to its contributions, also has some limitations. Therefore, our results should be evaluated within the framework of these limitations. First, our results were derived from a cross-sectional dataset from the PISA 2022 exam. This fact should be taken into account when making causal inferences. Since the data in cross-sectional studies are collected at a single point in time, the directionality of the relationships between variables cannot be determined with certainty. For this reason, our results should be interpreted as relational rather than causal inferences. In particular, we recommend that researchers conduct longitudinal studies to reveal the developmental nature of curiosity and imagination’s effects on reading. Our study only covers the Turkish sample. Therefore, this is another limitation of our study. Conducting similar studies—especially in a comparative manner with countries above and below the OECD average—will contribute to a better understanding of how student-level creative characteristics and school-level educational leadership and creativity-supporting conditions impact reading performance. Third, our study includes variables at the student and school levels. Variables from the PISA 2022 teacher questionnaire (i.e., use of creative pedagogy and teacher competence) could not be included in this study since this questionnaire was not administered in Türkiye. In this context, future research could fill this gap by using country/jurisdiction samples in which student, school, and teacher questionnaires were administered. Fourth, the instruments employed in this study were OECD-developed questionnaires. The operationalization of the constructs was therefore contingent upon the OECD’s conceptual framing and boundary specifications. Alternative conceptualizations and delimitations of these same constructs may exist in the broader literature. Consequently, subsequent research would benefit from examining these constructs through multiple definitional lenses, including those proposed by the OECD as well as alternative theoretical and empirical frameworks. Finally, the study may be limited by potential omitted variable bias. Important confounders, including students’ prior achievement, classroom practices, language background, regional differences, and school resources, were either unavailable or only partially controlled. Future studies should address this limitation by incorporating these variables into their analytical models to enhance the validity of the findings.

## Data Availability

Publicly available datasets were analyzed in this study. This data can be found at: https://www.oecd.org/en/data/datasets/pisa-2022-database.html.
